# Comparative Study on Hepatoprotective Effects of Traditional Herbs, Roots of *Angelica gigas* Nakai, *Glycyrrhiza uralensis* Fischer, *Zizyphus jujuba* Mill., and Fruits of *Paeonia lactiflora* Pall., on Ethanol-Induced Liver Injury in Mice

**DOI:** 10.3390/antiox13091137

**Published:** 2024-09-20

**Authors:** So-Yeon Kim, Kyung-Jin Oh, Yu-Ri Seo, Young-Woo Kim, Phil Hyun Song, Chang-Hyun Song

**Affiliations:** 1 Research Center for Herbal Convergence on Liver Disease, Gyeongsan 38610, Republic of Korea; yeon9925@gmail.com (S.-Y.K.); uree512@naver.com (Y.-R.S.); 2 Department of Urology, Chonnam National University Hospital, Chonnam National University Medical School, Gwangju 61469, Republic of Korea; exeokj@hanmail.net; 3 Department of Herbal Prescription, School of Korean Medicine, Dongguk University, Gyeongju 38066, Republic of Korea; ywk@dongguk.ac.kr; 4 Department of Urology, College of Medicine, Yeungnam University, Daegu 42415, Republic of Korea; 5 Department of Anatomy and Histology, College of Korean Medicine, Daegu Haany University, Gyeongsan 38610, Republic of Korea

**Keywords:** ALD, TCM, herb, Sirt1, CYP2E1, nrf2, antioxidant, anti-inflammatory, anti-apoptosis

## Abstract

Alcohol-associated liver disease (ALD) is a major cause of chronic liver disease, with few effective treatments besides alcohol abstinence. Angelicae Gigantis Radix (AG), Glycyrrhizae Radix et Rhizoma (GR), Paeoniae Radix (PR), and Zizyphi Fructus (ZF) are traditional herbs used to treat various ailments, including liver diseases. While several studies have reported the beneficial effects of GR on ALD, the effects of AG, PR, and ZF remain underexplored. Therefore, their efficacy and mechanisms against ALD were investigated using an alcohol-related liver injury model. The model was induced by ethanol gavage in C57BL/6J mice for 14 days, followed by oral administration of AG, GR, PR, and ZF one hour post-induction. The administration of these herbs reduced liver weight, and improved serum biomarkers of liver injury (ALT, AST, albumin). The herbs enhanced hepatic antioxidant capacity (GSH, SOD, catalase) and suppressed the production of proinflammatory cytokines (TNF-α, IL-1β) and apoptotic changes (caspase-3). The mechanisms of action involved lipid-lowering gene modulation through regulation of the cytochrome P450 2E1/Sirtuin 1/Nrf2 pathways. Histopathological and immunohistochemical analyses revealed that these herbs attenuated hepatocyte damage and steatosis via antioxidant, anti-inflammatory, and antiapoptotic effects. These findings suggest that traditional herbs, particularly AG, could be promising alternative therapies for treating ALD.

## 1. Introduction

Alcohol-associated liver disease (ALD) is a common liver disease with a high mortality rate, caused by excessive alcohol consumption [1]. ALD was responsible for 11 million deaths globally in 2019. With both prevalence and mortality rates rising annually, ALD has emerged as a major public health concern [1]. The disease initially manifests as hepatic steatosis and can progress to steatohepatitis, fibrosis, and cirrhosis, ultimately leading to end-stage hepatocellular carcinoma [2]. Hepatic steatosis is observed in over 90% of heavy drinkers and up to 35% of hospitalized patients with ALD [3]. Although hepatic steatosis can be clinically asymptomatic and reversible with alcohol abstinence, it is not an inert pathological change, with an annual mortality rate of 6% and a 10% risk of progression to cirrhosis over 10 years [4,5]. Since steatosis can induce metabolic changes that sensitize the liver to further injury, preventing steatosis may offer effective therapies to mitigate the progression of ALD [6]. However, there are currently no effective treatments to halt or reverse the disease’s progression, besides alcohol abstinence. The pathogenesis of ALD involves an imbalance in lipid metabolism, characterized by suppressed β-oxidation of fatty acids (FAs) and elevated lipogenesis [5]. Alcohol metabolism is generally coupled with the generation of its toxic metabolites (i.e., acetaldehyde) and reactive oxygen species (ROS), which promote lipid peroxidation, lipid synthesis, and hepatocellular injury [5,7]. Oxidative stress can be exacerbated by the depletion of antioxidants, leading to mitochondrial dysfunction and apoptosis in hepatocytes [6,8]. This triggers inflammatory responses, producing proinflammatory cytokines such as tumor necrosis factor (TNF)-α and interleukin (IL)-1β, which, in turn, worsen oxidative stress and apoptosis [9]. Thus, there is an urgent need for novel targeted therapies based on a thorough understanding of the development and progression of ALD.

Anti-inflammatory glucocorticoids and pentoxifylline are commonly used in patients with acute or severe alcoholic hepatitis [6,10]. However, glucocorticoids are ineffective in some patients and unsuitable for long-term treatment due to significant side effects and numerous contraindications. The efficacy of pentoxifylline also remains controversial [11,12,13]. Antioxidants (e.g., S-adenosylmethionine, N-acetylcysteine) and antifibrotic drugs (e.g., angiotensin receptor blockers, peroxisome proliferator-activated receptor [PPAR] agonists) have shown promise in preclinical studies, though their effectiveness has been inconsistent in clinical trials or requires further investigation [6]. Given the limited availability of effective treatments for ALD, there is growing interest in developing therapies based on natural extracts, which generally have fewer side effects and lower toxicity [14,15]. Accumulating evidence indicates that several natural compounds (e.g., silymarin, curcumin, anthocyanins, berberine) can effectively inhibit ALD progression by regulating multiple pathways involved in lipid metabolism, oxidative stress, inflammation, apoptosis, and the intestinal microbiota [8,15]. Additionally, several dietary phytochemicals rich in polyphenols and flavonoids have demonstrated effectiveness in animal models of ALD and toxicant-associated fatty liver disease [4,15,16]. Among these, silymarin is commonly used as a treatment option for liver diseases; however, no natural products, including silymarin, have received approval from the US Food and Drug Administration (FDA) as a drug for liver diseases, and further clinical validation of their efficacy is necessary [17,18].

Conversely, traditional herbal medicines prescribed for various ailments in East Asia have long-standing clinical records supporting their efficacy and safety, while there is a lack of scientific validation of their therapeutic effects. The hepatoprotective function of the Ayurvedic polyherbal formulation (Liv-52) is, as of recently, well recognized, with phase IV clinical trials showing it to be well-tolerated and effective in improving chronic liver disease [19]. Furthermore, many herbs have shown inhibitory effects on the progression of fatty liver diseases, suggesting the potential of traditional herbal medicines as alternative treatments for ALD [20]. Glycyrrhizae Radix et Rhizoma (GR; roots of *Glycyrrhiza uralensis* Fischer), Paeoniae Radix (PR; roots of *Paeonia lactiflora* Pall.), and Zizyphi Fructus (ZF; fruits of *Zizyphus jujuba* Mill.) are traditional tonifying herbs used to treat liver and inflammatory diseases [21,22,23,24]. Angelicae Gigantis Radix (AG; roots of *Angelica gigas* Nakai) is also a tonifying herb mainly prescribed for circulatory and gynecological diseases. These four herbs are considered non-toxic, and their bioactive components, including polyphenols, flavonoids, and polysaccharides, have been extensively analyzed [21,25,26,27,28]. Several studies have reported that GR and its compounds are effective in treating liver injury by inhibiting oxidative stress and inflammation in ethanol-induced animal models [29,30,31], and the clinical application of glycyrrhizin, a primary active compound of GR, has shown therapeutic potential for ALD [32]. The hepatoprotective effects of AG, PR, and ZF have been reported in animal models of different liver diseases with the following mechanisms: the inhibitory effects of AG on oxidative stress in an acute liver injury model induced by carbon tetrachloride (CCl_4_) [33] and on hepatic steatosis in a type 2 diabetes model [34]; the antioxidant and antiapoptotic effects of PR [35,36]; and the antioxidant and anti-inflammatory effects of ZF, in CCl_4_ or acetaminophen-induced models [37,38]. Although the anti-ALD effects of AG, PR, and ZF remain underexplored, their pharmacological properties, including inhibition of lipid accumulation, oxidative stress, and inflammation as well as their hepatoprotective effects, may contribute to the improvement of ALD. Therefore, to clarify the effectiveness of AG, GR, PR, and ZF on ALD, their efficacy was screened in a binge ethanol feeding model representing acute alcohol-induced liver injury, and the relevant mechanisms were examined [39].

## 2. Materials and Methods

### 2.1. Reagents

Silymarin and all other reagents, except those used for reverse transcription polymerase chain reaction (PCR) and immunoblotting assays, were obtained from Sigma-Aldrich (St. Louis, MO, USA). The four herbs, namely AG, GR, PR, and ZF (LOT Nos.: #ANGI2009, #GLUR2018, #PALA2013, and #ZIHO2016, respectively), are medicinal standard herbs provided and certified by the Korea FDA, and were prepared as described elsewhere [40,41,42,43]. AG, GR, PR, and ZF were collected from Bonghwa County (Korea), Ordos City (China), Sancheong County (Korea), and Gyeongsan City (Korea), respectively. The water-extracted herbs were filtered through two-layer mesh and Whatman No. 1 paper. In accordance with the Korean Pharmacopoeia, the herbs (with a purity of ≥99%) were standardized based on decursinol and decursin for AG, glycyrrhizin and liquiritin for GR, paeoniflorin for PR, and oleanolic acid for ZF as analytical markers of their bioactive components [21,25,26,27].

### 2.2. Free Radical Scavenging Activity

The 2,2-diphenyl-1-picrylhydrazyl (DPPH) assay was used to evaluate free radical scavenging activity, as described previously [44]. The herbal solutions and distilled water (DW) as the vehicle control were incubated with 0.4 mM DPPH in methanol for 30 min in the dark. Absorbance at 517 nm was determined using a microplate spectrophotometer (BIO-TEK, Winooski, VT, USA), and the free radical scavenging activity was calculated with the following Formula (1):

Activity (%) = [(absorbance of ‘A’ − absorbance of ‘B’)/absorbance of ‘A’] × 100, (1)

where “A” and “B” represent DW and herbal solutions, respectively.

### 2.3. Animals

The animal experiment was carried out in compliance with national regulations on the use and welfare of laboratory animals, with the protocol approved by the Institutional Animal Care and Use Committee of Daegu Haany University (Approval No. DHU2021-046). Six-week-old male C57BL/6J mice were obtained from Saeron Bio (Euiwang, Korea). The mice were housed four per polycarbonate cage in a controlled environment with a temperature of 20–25 °C, humidity of 40–55%, and a 12/12-h light/dark cycle. Food and water were provided ad libitum.

### 2.4. Ethanol-Induced Liver Injury Model and Treatments

After a one-week acclimatization period, the mice were divided into one normal group and six ethanol-induced liver injury model groups (*n* = 8 mice per group) based on body weight. The model was induced by oral gavage of ethanol at 5 g/kg once a day for 14 days, as described previously [45,46]. The normal group was administered an isocaloric maltose solution as the vehicle control for ethanol. One hour after induction, the normal group and one of the model groups were given DW orally as the normal and model controls, respectively. The remaining model groups received oral administration of silymarin at 250 mg/kg in DW and the four herbs (AG, GR, PR, and ZF) at 200 mg/kg in DW. The oral doses of silymarin and herbs were determined based on previous studies and clinical applications [31,33,36,38,45]. The oral gavage was administered at a dosage of 10 mL/kg. Animals were fasted overnight before the initial treatments and euthanasia to minimize dietary influences. Body weight was measured every day. One day after the final treatments, the mice were anesthetized under 2% isoflurane, and blood samples were collected. The mice were then euthanized using CO_2_ gas, and liver tissues were sampled for biochemical and histopathological analyses.

### 2.5. Blood Biochemistry

Blood samples were centrifuged at 10,000× *g* for 10 min at room temperature to collect the serum. The levels of alanine aminotransferase (ALT), aspartate aminotransferase (AST), and albumin in the serum were measured using a Beckman Coulter AU680 analyzer (Beckman Coulter, Indianapolis, IN, USA).

### 2.6. Assessments of Hepatic Antioxidant and Anti-Inflammatory Activities

A portion of the liver tissue (50 mg) was homogenized in phosphate-buffered saline (PBS) using a Taco^TM^ Prep Bead Beater (GeneReach Biotechnology Corp., Taichung, Taiwan) and an ultrasonic cell disruptor (KS-750, Madell Technology Corp., Ontario, CA, USA). The homogenates were centrifuged at 10,000× *g* for 10 min at 4 °C, and the supernatants were collected for analysis. Levels of malondialdehyde (MDA; #ab238537, Abcam, Waltham, MA, USA) and glutathione (GSH; #MBS267424, MyBioSource, San Diego, CA, USA), activities of superoxide dismutase (SOD) and catalase (#706002 and #707002, respectively, Cayman, Ann Arbor, MI, USA), and levels of TNF-α (#EM0183, FineTest, Wuhan, Hubei, China) and IL-1β (#MLB00C, R&D Systems, Minneapolis, MN, USA) were assessed using appropriate assay kits according to the manufacturer’s instructions. The reactions were measured at 540 nm for catalase and at 450 nm for the others under their respective standard curves using a microplate reader (BIO-TEK).

### 2.7. Real-Time Reverse Transcription PCR

Total RNA was extracted from liver tissue (10 mg) using TRIzol (Invitrogen, Carlsbad, CA, USA). The purity of the RNA was assessed by measuring the absorbance ratio from 260 nm to 280 nm using a spectrophotometer (BIO-TEK), and the RNA quantity was measured at 260 nm. The RNA (1 μg) was converted into complementary DNA (cDNA) using the ReverTra^TM^ Ace qPCR RT Master Mix (Toyobo, Osaka, Japan). The cDNA was then amplified using specific primers and TOPreal qPCR 2X preMIX (Enzynomics, Daejeon, Korea) on a CFX Connect Real-Time PCR Detection System (Bio-Rad, Hercules, CA, USA). The used primers are listed in Table 1. The PCR conditions were as follows: initial denaturation at 95 °C for 10 min, followed by 45 cycles of 95 °C for 10 s, 60 °C for 15 s, and 72 °C for 30 s. Gene expression was quantified relative to β-actin, and the relative expression levels were analyzed using the 2^–ΔΔCt^ method [47].

### 2.8. Immunoblotting

Liver homogenates in PBS were lysed in T-PER tissue protein extraction reagent (Thermo Scientific, Waltham, MA, USA) containing a 1× protease and phosphatase inhibitor cocktail (Quartett, Berlin, Germany) for 20 min on ice, followed by centrifugation at 10,000× *g* for 20 min at 4 °C. The total protein concentration in the supernatants was determined using a BCA assay kit (Thermo Scientific). The supernatants were then mixed with SDS-gel loading buffer (Bio-Rad) and boiled for 10 min. Equal amounts of protein samples were electrophoresed using Mini-PROTEAN TGX Stain-Free Gels (Bio-Rad) and transferred onto a nitrocellulose membrane using semi-dry blot transfer (Bio-Rad). The membrane was blocked with EveryBlot Blocking Buffer (Bio-Rad) for 10 min, and then incubated with primary antibodies overnight at 4 °C as follows: mouse antibody for sirtuin 1 (Sirt1; #8469, Cell Signaling, Danvers, MA, USA, 1:1000) and rabbit antibodies for nuclear factor erythroid 2-related factor (Nrf2; #PA5-27882, Thermo Scientific, 1:1000), cytochrome P450 2E1 (CYP2E1; #PA5-52652, Thermo Scientific, 1:1000), and cleaved caspase-3 (#9664, Cell Signaling, 1:1000). The membrane was then incubated with horseradish peroxidase (HRP)-conjugated goat secondary antibodies for mouse and rabbit IgG (#1706516 and #1706515, respectively, Bio-Rad, 1:1000) for 1 h. After antibody incubation, the membrane was washed five times with tris-buffered saline (TBS) containing 1% Tween 20 for 30 min. Protein expression was visualized using WesternBright^TM^ Sirius (Advansta, Menlo Park, CA, USA) and analyzed using a ChemiDoc instrument (Bio-Rad). The results were normalized against the levels of glyceraldehyde-3-phosphate dehydrogenase.

### 2.9. Histopathological Analysis

Liver samples were fixed in 10% neutral buffered formalin, and then paraffin-embedded or dehydrated in a 30% sucrose solution for frozen sections. The paraffin-embedded samples were cut into 3 μm sections and stained with hematoxylin and eosin (H&E). Frozen sections were serially cut at 10 μm and subjected to Oil red O or terminal deoxynucleotidyl transferase dUTP nick end labeling (TUNEL) stains using an assay kit (#ab206386, Abcam) according to the manufacturer’s instructions. Histopathological changes were examined in regions of interest (ROI) close to the hepatic central veins (zone 3), an area susceptible to alcohol-induced damage [48]. Analysis included the size of hepatocytes in 10 cells in H&E stains, the area stained with Oil red O, and the number of TUNEL-stained hepatocytes in 10 cells. Histomorphometric analyses were performed using NIS-Elements BR analysis software version 5.20 (Nikon, Tokyo, Japan) by a histopathologist blinded to the treatment groups.

### 2.10. Immunohistochemistry

Additional frozen sections were treated with 5% H_2_O_2_ in methanol for 15 min and blocked with 5% fetal bovine serum in TBS containing 0.3% Triton X-100 for 1 h. The sections were then incubated overnight at 4 °C with primary rabbit antibodies against nitrotyrosine (#A-21285, Invitrogen, 1:400), catalase (#ab16731, Abcam, 1:1000), TNF-α (#ab66579, Abcam, 1:600), and IL-1β (#ab205924, Abcam, 1:2000). They were then incubated with Dako EnVision system labeled polymer-HRP anti-rabbit secondary antibody (#K4003, DAKO, Kyoto, Japan) for 1 h. Immunoreactivity was visualized using the 3,3′-diaminobenzidine chromogen kit (#K3468, Dako) for 3 min, and counterstained with hematoxylin. Immunostains without the primary antibody were used as a negative control. The number of immunostained hepatocytes was counted in 10 cells within the ROI by a histopathologist blinded to the group assignments.

### 2.11. Statistical Analysis

Data were presented as means ± standard deviations for eight samples. The normality of the variables was assessed using the Kolmogorov–Smirnov test, and homogeneity of variance was evaluated using the Levene’s test. As the data showed normal distribution and homogeneity of variance, they were analyzed using one-way analysis of variance (ANOVA), followed by Tukey’s post hoc tests for multiple comparisons. In the DPPH assay, the measurements of the treatment groups were compared to the control group using Dunnett’s test. Kinetic body weight changes were analyzed using two-way ANOVA, with the groups and time-points as main factors, treating the time-points as repeated measures. A *p*-value of less than 0.05 was regarded as statistically significant.

## 3. Results

### 3.1. Free Radical Scavenging Activity of Traditional Herbs

**Figure 1:**
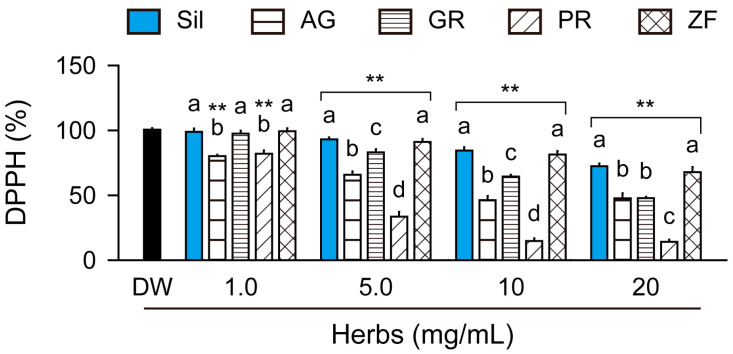
Significant free radical scavenging activity of herbs. Free radical scavenging activities of silymarin (Sil), Angelicae Gigantis Radix (AG), Glycyrrhizae Radix et Rhizoma (GR), Paeoniae Radix (PR), and Zizyphi Fructus (ZF) at the indicated concentrations were assessed using a 2,2-diphenyl-1-picrylhydrazyl (DPPH) assay. Values are presented as means ± standard deviations (SDs). **: *p* < 0.01, versus distilled water (DW) as the vehicle control, using Dunnett’s post hoc test. Different letters indicate significant differences between groups (*p* < 0.05, Tukey’s post hoc test).

Compared to DW as the vehicle control, the free radical scavenging activity was significantly dose-dependent for AG and PR at concentrations ranging from 1.0 to 20 mg/mL and for silymarin, GR, and ZF at concentrations ranging from 5.0 to 20 mg/mL (*p* < 0.01, Figure 1). The ranking score showed high free radical scavenging activities in the order of PR > AG > GR > ZF and silymarin (*p* < 0.05).

### 3.2. Changes in Body Weight and Liver Weight

**Figure 2:**
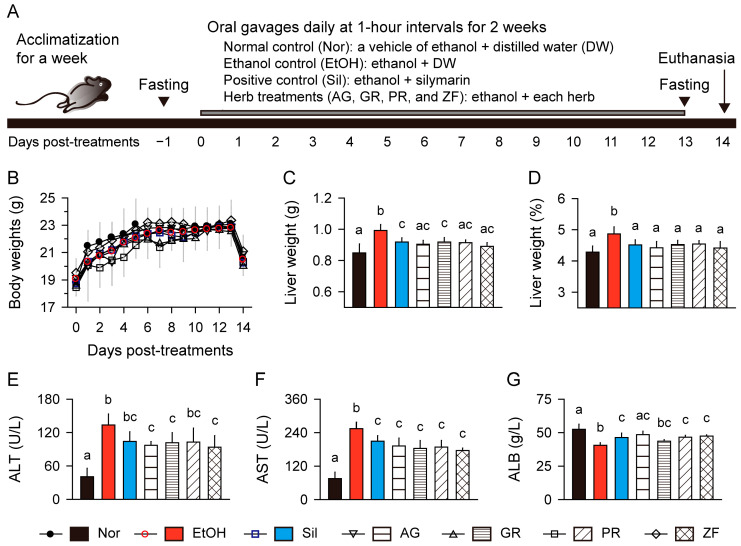
Improvements in body weight, liver weight, and serum biomarkers related to liver injury. (**A**) Schematic of the experimental design. (**B**) Body weight changes. (**C**) Absolute liver weight. (**D**) Relative liver weight to body weight. (**E**–**G**) Serum levels of alanine aminotransferase (ALT), aspartate aminotransferase (AST), and albumin (ALB). Values are presented as means ± SDs. Different letters indicate significant differences between groups (*p* < 0.05, Tukey’s post hoc test).

As described in Section 2.4 of the Materials and Methods, an ethanol-induced liver injury model was used, with silymarin, AG, GR, PR, and ZF administered orally for 14 days, while the normal and model control groups received DW (Figure 2A). Body weight changes were similar across all groups, irrespective of the treatments (Figure 2B). After all treatments, both absolute liver weight and relative liver weight to body weight were significantly increased in the model control group compared to the normal control (*p* < 0.01); however, they were reduced in the treatment groups of silymarin and herbs compared to the model control (*p* < 0.05, Figure 2C,D). The absolute liver weight in the AG, PR, and ZF groups was similar to that in the normal control group.

### 3.3. Improvements in Serum Biomarkers of Liver Injury

Serum levels of ALT and AST were increased in the model control group relative to the normal control (*p* < 0.01, Figure 2E,F). However, these levels were significantly reduced in the silymarin and herb groups compared to the model control (*p* < 0.05). Conversely, the albumin level was lower in the model control than in the normal control (*p* < 0.01), but it was higher in the silymarin and herb groups, except for the GR group, compared to the model control (*p* < 0.05, Figure 2G). The albumin level was similar between the normal control and AG groups.

### 3.4. Hepatic Antioxidant and Anti-Inflammatory Activities

**Figure 3:**
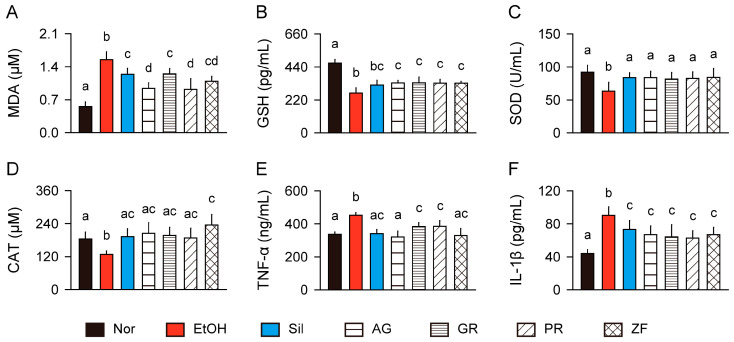
Activation of hepatic antioxidant and anti-inflammatory effects. (**A**) Levels of malondialdehyde (MDA). (**B**) Levels of glutathione (GSH). (**C**,**D**) Activities of superoxide dismutase (SOD) and catalase (CAT). (**E**,**F**) Levels of tumor necrosis factor (TNF)-α and interleukin (IL)-1β. Values are presented as means ± SDs. Different letters indicate significant differences between groups (*p* < 0.05, Tukey’s post hoc test).

In the model control group, compared to the normal control, the hepatic level of MDA was increased, while the GSH level and SOD and catalase activities were reduced (*p* < 0.01, Figure 3A–D). However, these parameters were significantly reversed in the silymarin and herb groups compared to the model control (*p* < 0.05). Notably, the inhibition of MDA levels was significantly greater in the AG and PR groups than in the silymarin and GR groups, and catalase activity was elevated in the ZF group compared to the normal control (*p* < 0.05). The model control group exhibited elevated levels of proinflammatory cytokines (TNF-α and IL-1β) compared to the normal control (*p* < 0.01). However, the proinflammatory cytokine levels were reduced in the treatment groups of silymarin and herbs compared to the model control (*p* < 0.05, Figure 3E,F). The inhibitory effects on TNF-α levels were stronger in the AG group compared to the GR and PR groups (*p* < 0.05).

### 3.5. Signaling Pathway Related to Alcohol Metabolism in the Liver

**Figure 4:**
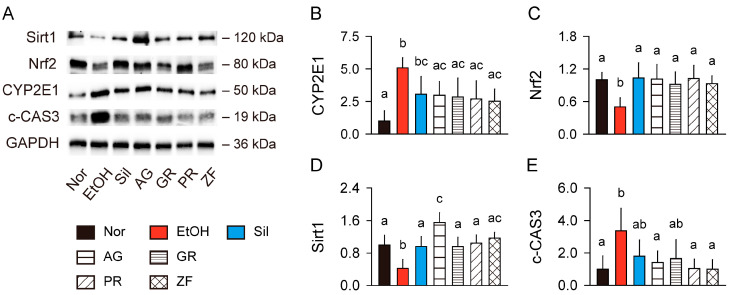
Regulation of alcohol metabolism-related signaling pathways in the liver. (**A**) Representative immunoblots for cytochrome P450 2E1 (CYP2E1), nuclear factor erythroid 2-related factor (Nrf2), sirtuin 1 (Sirt1), and cleaved caspase-3 (c-CAS3). Expression levels were normalized to glyceraldehyde-3-phosphate dehydrogenase (GAPDH). (**B**–**E**) Relative expression levels of CYP2E1, Nrf2, Sirt1, and c-CAS3. Values are presented as means ± SDs. Different letters indicate significant differences between groups (*p* < 0.05, Tukey’s post hoc test).

The hepatic expressions of CYP2E1, Nrf2, Sirt1, and cleaved caspase-3 were assessed for ALD-related signaling pathways (Figure 4) [13,15]. The expression of CYP2E1 was increased in the model control group compared to the normal control (*p* < 0.01), but reduced in the silymarin and herb groups compared to the model control (*p* < 0.05, Figure 4A,B). Conversely, the expressions of Nrf2 and Sirt1 were reduced in the model control group compared to the normal control (*p* < 0.01); however, they were increased in the treatment groups of silymarin and herbs compared to the model control (*p* < 0.05, Figure 4C,D). Notably, the hepatic Sirt1 levels were significantly increased in the AG group compared to the silymarin, GR, and PR groups (*p* < 0.05). The expression of cleaved caspase-3 was increased in the model control group compared to the normal control (*p* < 0.01); however, it was reduced in the treatment groups compared to the model control (*p* < 0.05, Figure 4E).

### 3.6. Gene Regulation Related to Lipid Metabolism in the Liver

**Figure 5:**
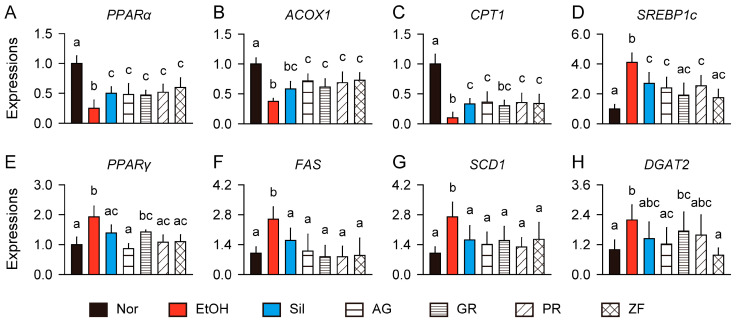
Gene regulation related to the inhibition of hepatic lipid accumulation. (**A**–**C**) Relative expression levels of lipid catabolism-related genes, peroxisome proliferator-activated receptor (PPAR)α, acyl-Coenzyme A oxidase 1 (ACOX1), and carnitine palmitoyltransferase 1 (CPT1). (**D**–**H**) Relative expression levels of lipid anabolism-related genes, sterol regulatory element-binding protein 1c (SREBP1c), PPARγ, fatty acid synthase (FAS), stearoyl-Coenzyme A desaturase 1 (SCD1), and diacylglycerol O-acyltransferase 2 (DGAT2). Values are presented as means ± SDs. Different letters indicate significant differences between groups (*p* < 0.05, Tukey’s post hoc test).

The lipid metabolism-regulating effects were evaluated by analyzing gene expressions for PPARα and its target genes, acyl-Coenzyme A oxidase 1 (ACOX1) and carnitine palmitoyltransferase 1 (CPT1), which are involved in lipid catabolism [5]. Additionally, gene expressions for sterol regulatory element-binding protein 1c (SREBP1c), PPARγ, fatty acid synthase (FAS), stearoyl-Coenzyme A desaturase 1 (SCD1), and diacylglycerol O-acyltransferase 2 (DGAT2) were assessed as lipid anabolic genes (Figure 5). The lipolytic genes, PPARα, ACOX1, and CPT1, were downregulated in the model control group compared to the normal control, while the lipogenic genes, SREBP1c, PPARγ, FAS, SCD1, and DGAT2, were upregulated (*p* < 0.01, Figure 5A–H). However, compared to the model control, the lipolytic genes were upregulated in the treatment groups of silymarin and herbs, with the exception of *CPT1* in the GR group (*p* < 0.05). Meanwhile, the lipogenic genes, except for *DGAT2*, were downregulated in the silymarin and herb groups compared to the model control (*p* < 0.05). *DGAT2* was downregulated only in the AG and ZF groups compared to the model control (*p* < 0.05).

### 3.7. Histopathological Improvements in the Liver

**Figure 6:**
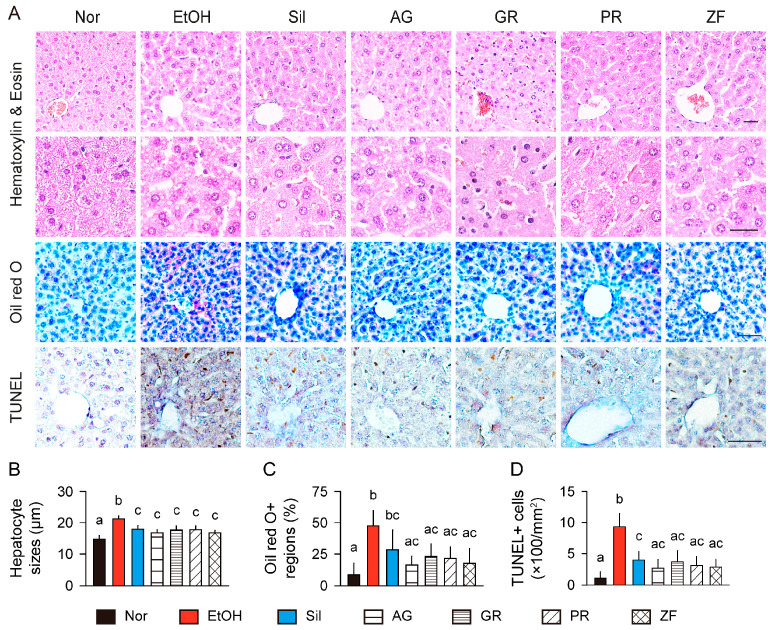
Histopathological improvements in liver injury and steatosis. (**A**) Representative images of regions of interest close to the hepatic central veins stained with hematoxylin and eosin (H&E), Oil red O, and terminal deoxynucleotidyl transferase dUTP nick end labeling (TUNEL). Scale bars = 50 μm. (**B**) Hepatocyte sizes in H&E stains. (**C**) Oil red O-stained regions. (**D**) Numbers of hepatocytes stained with TUNEL. Values are presented as means ± SDs. Different letters indicate significant differences between groups (*p* < 0.05, Tukey’s post hoc test).

The ethanol-induced liver injury and fat accumulation were observed in H&E, Oil red O, and TUNEL stains (Figure 6). Hepatocyte swelling, apoptosis, and lipid accumulation were prominent in the model control, but these changes were notably reduced in the silymarin and herb groups (Figure 6A). The model control group showed increased hepatocyte sizes, Oil red O-stained regions, and numbers of TUNEL-stained hepatocytes compared to the normal control (*p* < 0.01). However, these parameters were significantly reduced in the silymarin and herb groups relative to the model control (*p* < 0.05, Figure 6B–D). The Oil red O-stained regions and numbers of TUNEL-stained hepatocytes in the herb groups were similar to those of the normal control group.

### 3.8. Hepatic Antioxidant and Anti-Inflammatory Effects in Immunohistochemistry

**Figure 7:**
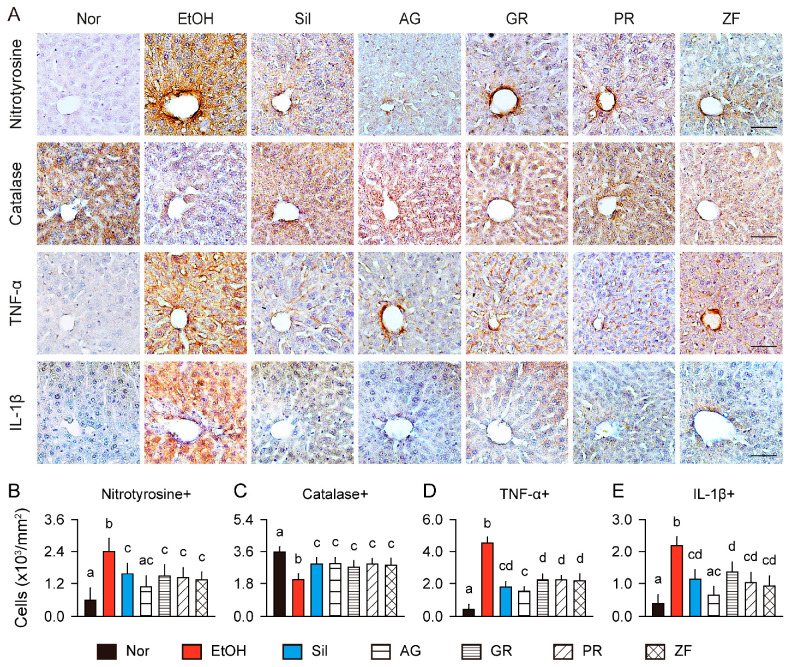
Enhanced antioxidant and anti-inflammatory effects in immunohistochemical analysis of the liver. (**A**) Representative images of regions of interest close to the hepatic central veins immunostained for nitrotyrosine, catalase, TNF-α, and IL-1β. Scale bars = 50 μm. (**B**–**E**) Numbers of immunostained hepatocytes. Values are presented as means ± SDs. Different letters indicate significant differences between groups (*p* < 0.05, Tukey’s post hoc test).

The antioxidant and anti-inflammatory effects of the herbs were assessed in immunostains for nitrotyrosine, catalase, TNF-α, and IL-1β (Figure 7). The model control group exhibited higher numbers of nitrotyrosine-, TNF-α-, and IL-1β-positive cells compared to the normal control (*p* < 0.01), while the numbers of catalase-positive cells were reduced (Figure 7A–E). However, these trends were notably reversed in the silymarin and herb groups relative to the model control (*p* < 0.05). The numbers of nitrotyrosine- and IL-1β-positive cells were similar between the normal control and AG groups. Moreover, the AG group showed significant reductions in the numbers of TNF-α-positive cells compared to the GR, PR, and ZF groups, and reductions in the number of IL-1β-positive cells compared to the GR group (*p* < 0.05).

## 4. Discussion

The current binge ethanol feeding model exhibited increases in liver weight and serum levels of ALT and AST, along with histopathological features such as hepatocyte fat accumulation and ballooning degeneration, consistent with previous studies [39,45,46]. The ethanol exposure also resulted in an AST/ALT ratio greater than two and reduced albumin levels, mirroring symptoms observed in patients with ALD at the stage of simple steatosis to steatohepatitis [49]. However, oral administration of AG, GR, PR, and ZF at 200 mg/kg (approximately 2 g/kg for a 60 kg adult) reduced liver weight and improved serum biomarkers of liver injury through lipid-lowering, antioxidant, anti-inflammatory, and antiapoptotic mechanisms. Histopathological analyses revealed that these herbal treatments attenuated ethanol-induced liver injury and steatosis. This screening study is the first to compare the therapeutic potential of AG, GR, PR, and ZF in an ethanol-induced ALD model. GR, PR, and ZF are traditionally used for treating liver diseases, while AG is mainly used as a hematopoietic or anti-inflammatory agent [21,22,23,24]. Additionally, a previous study reported that the same oral dose of GR (200 mg/kg) for 4 weeks restores alcohol-induced liver injury via antioxidant and anti-inflammatory effects in a liquid alcohol diet mouse model [31]. However, among these herbs, AG was the most significantly effective in improving ethanol-induced liver injury. These results suggest that the effectiveness of the herbs in ameliorating ethanol-induced liver injury can be ranked as follows: AG ≥ ZF ≥ silymarin and PR ≥ GR.

Alcohol metabolism is driven by a complex interplay between oxidative stress, inflammation, and apoptosis, creating a vicious cycle that perpetuates liver damage [6]. Here, ethanol exposure increased lipid peroxidation (MDA and nitrotyrosine) and depleted antioxidants (GSH, SOD, and catalase), probably due to the activation of the CYP2E1 enzymes. It is likely that the consequent overproduction of ROS and free radicals increased the production of proinflammatory cytokines (TNF-α and IL-1β) and apoptotic changes (caspase-3, TUNEL stain) [50]. Indeed, ROS is known as a crucial initiator in the progression of acute alcohol-induced toxicity [51]. Although free radical scavenging activity was lower in silymarin and ZF than in other herbs, all treatments of the herbs and silymarin enhanced hepatic antioxidant defense capacity and reduced the production of proinflammatory cytokines and apoptosis, through the regulation of CYP2E1 and Nrf2 signaling pathways. Decursin, decursinol angelate, and decursinol are the primary active compounds of AG and have shown antinociceptive properties in various inflammatory pain models (reviewed in [52]). Decursin from AG has been shown to activate the AMP-activated protein kinase (AMPK) pathway exerting as a positive influence on Nrf2 signaling, in a HepG2 injury model induced by arachidonic acid with iron, resulting in inhibition of ROS production, mitochondrial dysfunction, and apoptosis [33]. Various phenolic compounds and flavonoids from ZF have demonstrated cytoprotective activity against alcohol-induced oxidative stress through the upregulation of Nrf2-mediated antioxidant defense enzymes [43], as well as hepatoprotective effects against acetaminophen-induced liver injury [53]. Moreover, antioxidant effects through the activation of the Nrf2 pathway have been reported in the active components of GR, including liquiritin, liquiritigenin, and glycycoumarin, resulting in reduced drug toxicity [54] and contributing to hepatoprotective activity in chronic or acute ethanol exposure [29]. Paeoniflorin from PR has demonstrated not only anti-inflammatory effects in a traumatic stress animal model [55], but also protective mechanisms against cholestasis through Nrf2-dependent antioxidant efficacy in a cholestatic liver injury model [56]. However, the present results indicate that the AG and ZF groups exhibited superior efficacy in suppressing lipid peroxidation and the production of pro-inflammatory cytokines in the liver, which may be attributed to their comprehensive effects of inhibiting oxidative stress, inflammation, and apoptosis caused by alcohol metabolism. Since these beneficial effects can be attributed to specific components of the herbs, further research is needed to elucidate the precise molecular mechanisms of the key active components in the medicinal herbs used.

No effective treatments currently exist for fatty liver diseases, despite the focus on reducing lipid accumulation as a primary target in the development of pharmaceutical agents for the early stages of ALD. In alcohol-associated hepatic steatosis, ROS-induced oxidative stress may suppress energy metabolism signaling pathways linked to AMPK and Sirt1, while promoting the production of TNF-α [5,57]. Inhibition of the Sirt1 and AMPK pathways activates SREBP1c and PPARγ, leading to the upregulation of lipogenic genes (FAS, SCD1, and DGAT). In this context, AMPK, Sirt1, SREBP1c, and PPARα signaling pathways have been highlighted as major molecular targets for novel therapeutic agents aimed at inhibiting the progression of hepatic steatosis [6,20]. In this study, the model showed reduced hepatic expression of Sirt1, coupled with the upregulation of lipogenic genes (SREBP1c, PPARγ, FAS, SCD1, and DGAT2). The accumulation of free FAs and triglycerides may be linked to the downregulation of genes involved in β-oxidation (PPARα, ACOX1, and CPT1). However, treatments with these herbs increased Sirt1 expression, and reversed the abnormal gene expressions related to the imbalance in lipid metabolism, except for *DGAT2*, which was downregulated only in the AG and ZF groups, probably due to their superior activation of the Sirt1 pathway. Moreover, considering that TNF-α promotes hepatic fat accumulation by increasing the hepatic uptake of exogenous free FAs, facilitating the incorporation of FAs into triglycerides, and inhibiting FA β-oxidation, the significant anti-TNF-α effects of AG may contribute to restoring lipid balance in ethanol-induced dysregulation of hepatic lipid metabolism [50]. Both AG and its compound, decursin, have been shown to improve glucose tolerance, inflammation, and steatosis in a high-fat diet animal model [58], and the clinical application of AG extract has been reported to improve blood triglyceride levels and prevent hypertriglyceridemia [59]. This suggests that AG may also have potential in the prevention and treatment of alcohol-associated fatty liver disease.

Clinical trials in patients with alcohol-associated hepatitis are ongoing for some targeted agents (e.g., IL-22 agonist, DNA hypermethylation inhibitor, anti-ROS) with promising results [13,60]. However, most have failed to demonstrate significant benefits through single therapeutic mechanisms aimed at inhibiting specific inflammation (e.g., inhibitors of TNF or IL-1 receptor) and apoptosis (e.g., emricasan, selonsertib) or stimulating liver regeneration (e.g., granulocyte colony-stimulating factor). Metadoxine, an antioxidant drug used to treat acute alcoholism, improves liver function indirectly by accelerating alcohol excretion, but does not improve liver steatosis or inflammation [15]. This underscores the need for treatments with multiple pharmacological mechanisms to address the complex pathogenesis of ALD. In this study, the oral administration of AG, GR, PR, and ZF, demonstrated potential anti-ALD effects by targeting multiple mechanisms, including lipid metabolism, oxidative stress, inflammation, and apoptosis, through the inhibition of CYP2E1 and activation of Sirt1 and Nrf2 pathways. The therapeutic efficacy of these herbs was comparable to or better than that of the silymarin group, with the AG and ZF groups showing particularly significant anti-inflammatory and lipid-lowering effects. However, this study is limited by the use of a single oral dose of 200 mg/kg administered for 14 days in a binge ethanol-induced acute liver injury model. Therefore, further studies are needed to determine the optimal oral dosage of these herbs based on component analysis and to validate their efficacy in chronic ALD animal models and subsequent well-designed clinical trials. Additionally, pharmacokinetic analysis of the herbs, along with assessments of side effects and toxicity, is necessary to evaluate the potential synergistic effects of combined herbal candidates, considering the composition of traditional polyherbal formulas.

In traditional Chinese medicine (TCM) and Korean medicine, liver disease is perceived to be caused by poor blood circulation, toxin accumulation, and energy deficiency. Traditional tonifying herbs are prescribed to promote the circulation of blood and Qi, and to tonify the liver in ALD patients [61]. AG and PR are classified as blood-tonifying herbs, while GR and ZF are considered Qi-tonifying herbs. GR, PR, and their combination are frequently prescribed to treat metabolism-associated fatty liver diseases in TCM [22]. It has been reported that AG and its compounds (decursin and decursinol angelate) have no subchronic and subacute toxicities, respectively, in rats at oral doses of up to 2 g/kg [28,62]. AG extracts or herbal mixtures containing AG are commercially available as dietary supplements for the relief of pain and postmenopausal symptoms globally [63], and ESP-102, an extract from AG, *Saururus chinensis*, and *Schisandra chinensis*, is used as an herbal medicine and health-promoting supplement in Korea [64]. Additionally, GR (licorice), PR (peony), and ZF (jujube) are generally considered non-toxic and are widely used as safe edible or medicinal herbs [21,26,27]. This suggests that these traditional herbal medicines may serve as valuable resources that could be effectively linked to clinical trials for the development of novel drugs for ALD. Furthermore, Angelicae Sinensis Radix (Chinese Danggui), similar to AG (Korean Danggui), is not only valued for its benefits in pain relief and gynecologic health, but is also one of the top 10 herbs frequently used in treating liver cirrhosis in TCM [65]. Treatments with polysaccharides from *Angelicae sinensis* have also shown improvements in serum biomarkers in an ALD animal model, with significant CYP2E1-mediated antioxidant and lipid-balancing effects [66]. The present results provide insights into the potential of traditional herbs, particularly AG, as alternative therapies for treating ALD.

## Figures and Tables

**Table 1:** Primers for quantitative reverse transcription polymerase chain reaction.

Targets (GenBank IDs)	Sequence (5′–3′)
ACOX1 (NM_013495)	Forward: GAATCAGGGCACCACTGCTCA
	Reverse: CCTCGAAGATGAGTTCCGTGG
CPT1 (NM_013495)	Forward: TGCATACCAAAGTGGACCCC
	Reverse: ACGCCACTCACGATGTTCTT
FAS (NM_0079883)	Forward: GCTTCGCCAACTCTACCATG
	Reverse: CCATCGCTTCCAGGACAATG
DGAT2 (NM_026384)	Forward: AGTGGCAATGCTATCATCATCGT
	Reverse: AAGGAATAAGTGGGAACCAGATCA
PPARα (NM_001113418)	Forward: TGCCTTCCCTGTGAACTGAC
	Reverse: CCATGTTGGATGGATGTGGC
PPARγ (NM_001127330)	Forward: ACGCGGAAGAAGAGACCTGG
	Reverse: AGTGTGACTTCTCCTCAGCC
SCD1 (NM_009127)	Forward: TGGAGACGGGAGTCACAAGA Reverse: CCCCGATAGCAATATCCAGTTG
SREBP1c (NM_011480)	Forward: GATGTGCGAACTGGACACAG Reverse: CATAGGGGGCGTCAAACAG
β-actin (NM_007393)	Forward: CAGCAAGCAGGAGTACGATGA
	Reverse: AACGCAGCTCAGTAACAGTCC

ACOX1, acyl-Coenzyme A oxidase 1; CPT1, carnitine palmitoyltransferase 1; FAS, fatty acid synthase; DGAT2, diacylglycerol O-acyltransferase 2; PPAR, peroxisome proliferator-activated receptor; SCD1, stearoyl-Coenzyme A desaturase 1; SREBP1c, sterol regulatory element-binding protein 1c.

## Data Availability

Data are contained within the article.
